# Assessing the Compliance of Dental Clinicians towards Regulatory Infection Control Guidelines Using a Newly Developed Survey Tool: A Pilot Cross-Sectional Study in India

**DOI:** 10.3390/healthcare10101877

**Published:** 2022-09-26

**Authors:** Pragati Kaurani, Kavita Batra, Himangini Rathore Hooja, N. Gopi Chander, Anamitra Bhowmick, Suraj Arora, Suheel Manzoor Baba, Shafait Ullah Khateeb, Anshad M. Abdulla, Vishakha Grover, Priyanka Saluja

**Affiliations:** 1 Department of Prosthodontics, Mahatma Gandhi Dental College and Hospital, Sitapura Industrial Area, Jaipur 302004, India; 2 Department of Medical Education, Kirk Kerkorian School of Medicine, University of Nevada, Las Vegas, NV 89102, USA; 3 Office of Research, Kirk Kerkorian School of Medicine, University of Nevada, Las Vegas, NV 89102, USA; 4 Department of Psychology, IIS (deemed to be University), Gurukul Marg, Mansarovar, Jaipur 302020, Rajasthan, India; 5 Department of Prosthodontics, SRM Dental College, Ramapuram Chennai 600089, India; 6 Indegene Lifesystems Private Limited, Bengaluru, Karnataka 560045, India; 7 Department of Restorative Dental Sciences, College of Dentistry, King Khalid University, Abha 61321, Saudi Arabia; 8 Department of Pediatric Dentistry & Orthodontics, College of Dentistry, King Khalid University, Abha 61321, Saudi Arabia; 9 Department of Periodontology & Oral Implantology, Dr. H. S. J. Institute of Dental Sciences & Hospital, Panjab University, Chandigarh 160015, India; 10 Department of Conservative Dentistry and Endodontics, JCD Dental College, Vidyapeeth Sirsa 125055, Haryana, India

**Keywords:** SARS-CoV-2, COVID-19, dental guidelines, dental clinicians, exploratory factor analysis

## Abstract

Adherence to the dental practice regulatory guidelines instituted during the COVID-19 pandemic is essential to minimize the transmission of SARS-CoV-2 strains. Given the lack of a valid and reliable survey tool to assess the adherence to dental practice guidelines, this study aims to develop, validate, and test a survey tool on a pilot sample of dental clinicians practicing in India. A survey tool was developed/validated through a sequential phasic approach: Phase I- developing survey using conceptual and literature framework; Phase II: ascertaining its validity and reliability; Phase III: pilot testing; and Phase IV: assessing construct validity by exploratory factor analysis (EFA) on the responses collected in Spring 2021. The EFA was achieved using a traditional unweighted least squares extraction method through a varimax rotation with Kaiser normalization. A six-factor solution with 18 items (with the global reliability of 86%) related to screening, regular infection prevention measures, infection control inside the dental operatory, disinfection of the dental unit, disposal, and other COVID-19-specific preventive measures were extracted. Our sample had higher compliance with regard to providing alcohol-based hand scrubs, providing protective gear to attendees, collecting travel/medical history, and screening patients for COVID-19 symptoms. In contrast, less compliance was observed regarding the use of paperless forms of practice and rubber dams in the operatory. The use of a validated survey tool ensures the collection of reliable and valid data, which can serve as baseline data to measure the uptake and effectiveness of dental practice regulatory guidelines in a clinical setting and community dental health clinics.

## 1. Introduction

Globally, since the inception of the COVID-19 pandemic, several efforts to control the disease have collectively been made. Social distancing, the development of vaccines, and various clinical trials of drugs to treat severe acute respiratory syndrome coronavirus 2 (SARS-CoV-2) infection are among the different measures that continue to be implemented worldwide [1,2,3]. However, to date, the virus and its mutants continue to infect thousands of people [4,5].

Several considerations of various workplaces that could potentially transmit the disease were made based on the research findings and observations. Among these work settings, dental operatories gathered attention as a potential environment to increase the transmission of the virus through dental procedures. Many routine dental procedures are aerosol-generating dental procedures (AGDPs) that could undoubtedly be the potential source of spread of SARS-CoV-2 in dental settings [6]. Moreover, due to the close proximity of dental care workers (DCWs) and patients during dental procedures, the risk of transmission to and from patients is inevitable, which underscore the need to follow stringent infection control practices in these risky work settings [7,8]. Due to these risks, dental practice was adversely affected across the globe during the early stages of the pandemic [9].

To minimize the spread of infection and still restore dental practice, strict disinfection and protection protocols were needed. It became the focal point of research and with the available evidence, governing dental bodies across the globe issued guidelines and standard operating procedures (SOPs) that would help to curb transmission. Various organizations and governing bodies across several countries, such as the American Dental Association (ADA) and the Centers for Disease Control and Prevention (CDC), World Health Organization (WHO), European Center for Disease Prevention and Control, and Canadian Dental Association, released guidelines for dental practice during the pandemic [10,11,12,13,14,15]. Similarly, the dental governing body in India, i.e., the Dental Council of India (DCI), initially released its first set of advisories, followed by the issuing of the Dental Clinics Protocol, covering vital aspects of infection control that were deemed necessary to control the spread of infection [16]. The guidelines covered all important aspects of infection control, such as measures to be taken during clinical work, personal protective equipment (PPE) and their safe disposal, disinfection of surfaces, and all other relevant aspects that would minimize the spread of infection and provide protection to clinical staff and dental patients [17,18].

These guidelines and protective measures formed the “new reality” that helped to maintain the continuum of clinical practice using precautionary measures to limit the spread [9]. However, the efficacy and impact of the guidelines depends on the compliance of the dental fraternity in following a given set of guidelines. In other words, merely the dissemination of guidelines does not necessarily guarantee their implementation in a clinical scenario [19]. Previous reports indicated that adherence to preventive measures and protective behaviors was associated with knowledge and risk perception of SARS-CoV-2 [17,20,21,22]. In India, reports indicated that knowledge about the COVID-19 pandemic among dentists ranged from satisfactory to notable [23,24]. Retrospectively, during the pre-COVID era, questionnaires and direct observations were used to assess the uptake of infection control recommendations among dentists [25,26,27]. However, to date, studies to assess the uptake of these guidelines among Indian dentists during the COVID-19 pandemic period remains an area of possible exploration.

A few studies have attempted to measure their adherence; however, the entire breadth of the regulatory guidelines was not accounted for when assessing compliance. In one study, the adherence of Vietnamese dental care workers (DCWs) to COVID-19-preventive measures was assessed from 1 August to 9 September 2021 using six questions that covered aspects, such as wearing personal protective equipment (PPE), use of masks during patient care, hand hygiene, cleaning and disinfection of surfaces during patient care, safe disposal of waste, and other procedures to prevent the transmission of saliva using a Likert scale that ranged from “never (0)” to “always (4)” [28]. In another study, the adherence of dental practitioners to the health regulations in Israel was assessed between 17 March 2020 and 30 April 2020 using an online questionnaire and found that only 68.93% of dentists were compliant to all the given recommendations [29]. However, not much is known regarding the adherence of dental clinicians to the guidelines for dental practice provided by the DCI during the pandemic. Moreover, a validated survey tool to assess the level of adherence of dental clinicians towards the guidelines issued by the governing dental body in the Indian context is lacking. Such a tool can help to assess the efforts made by dental practitioners to ensure best practice and management approaches to prevent the spread of infection during the pandemic. Therefore, the aim of this study was to develop, validate, and pilot test a questionnaire or survey tool that can measure the compliance of dental practitioners towards the guidelines for dental practice issued by the DCI.

## 2. Materials and Methods

### 2.1. Study Design 

The current study was a cross-sectional pilot study conducted in sequential phases to develop, validate, and pilot test a questionnaire to evaluate the adherence to COVID-19 protocols for dental practice as mandated by the DCI among dental health practitioners. Phase I consisted of the process of item generation and development of the instrument/questionnaire, Phase II consisted of ascertaining the validity and reliability of the instrument, and Phase III consisted of pilot testing the developed questionnaire. The last phase, Phase IV, involved item purification by exploratory factor analysis (construct validity).

### 2.2. Ethical Considerations

The research protocol for the current study was approved by the Institutional Ethics Committee of SRM Dental College (dated 11/21/2020 SRMU/M&HS/SRMDC/2020/S/031). Voluntary participation was ensured, and electronic informed consent was obtained. The confidentiality of the participants’ responses was ensured.

### 2.3. Development and Validation of a Survey Instrument

The new instrument was developed by a team of three authors, a practicing dentist (P.K.), public health researcher (K.B.), and a behavioral science researcher (H.R.H.). In addition, semi-structured interviews were conducted with practicing dental specialists who were familiar with the practice guidelines to develop the concepts used in the tool-building process. All steps taken to assess the psychometric validity of the survey tools are described below. 

#### 2.3.1. Phase I: Development of the Instrument

A thorough literature search using PubMed and Google Scholar was performed to provide a foundation to build upon in relation to the dental practice guidelines. Articles were searched using the keywords “dental practice”,” COVID-19 guidelines”, “prevention of spread of infection”, “COVID-19 dental practice”, “hand disinfection”, “dental clinics”, and “SARS-COV 2”. A combination of appropriate Boolean operators (AND, OR), truncation, and the MeSH terms was used. Further, articles based on guidelines laid out by different health organizations across the world with regard to dental practice during the COVID-19 pandemic in India and in other countries were retrieved to create a literature matrix. A further literature search was caried out on (1) infection control in a dental operatory during the pandemic, (2) screening and appointment of dental patients during the pandemic, and (3) personal protection of dental clinicians. Articles were selected that reviewed and/or provided detailed information on the infection control protocols of dental practices during the COVID-19 pandemic. Additionally, the Dental Clinics Protocol, as issued by the DCI, was thoroughly reviewed for inclusion in the literature matrix [15]. 

Based upon the thorough literature searches and interviews, the team created the first preliminary version of the questionnaire. All items were refined and organized in a suitable format in order to attain a usable form. Subsequently, relevant items were identified and reviewed for structure and clarity. The length of each item was kept as short as possible without affecting its comprehensibility. To avoid acquiescent response bias, a mixture of both positively and negatively worded items were used. Leading questions, double negatives or double-barreled questions were avoided [30,31]. Any ambiguous wordings were modified and an initial version of a 30-item questionnaire was created. The responses were measured on a 5-point Likert Scale, which referred to “how often” the practitioners followed a particular guideline during the pandemic (always, often, sometimes, rarely, never). Further, a basic demographic section was added towards the end of the questionnaire.

#### 2.3.2. Phase II: Validation of the Instrument: The Validation of the Instrument Was Performed in Two Steps, as Follows

A. Content Validity

Content validity was assessed in two rounds. For the first round, 7 experts (consisting of a prosthodontist, oral radiologist, public health researcher, endodontist, and a dental hygienist) were asked to evaluate and validate the contents. The investigators of the study provided the experts with a cover letter explaining the reasons for inviting the expert to participate. Experts were supplied with guidelines provided by the DCI, along with developed instruments and clear instructions on how to rate each item. The experts were asked to independently rate the relevance of each item using a 4-point Likert scale (1 = not relevant, 2 = somewhat relevant, 3 = quite relevant, and 4 = very relevant). In cases where no response was obtained, a reminder email was sent after a week. The content validity index (CVI) was used to estimate the validity of the individual items (Item–Content Validity Index—I-CV1) and scale (Scale–Content Validity Index—S-CVI) and, based on the results, modifications were made iteratively [32]. For the second phase of the content validation, the modified instrument was circulated among 10 different cohorts of experts and the same process was repeated. On the basis of the rating of experts and practitioners, the I-CVI and S-CVI were computed [33,34].

B. Face Validity

The face validity of the questionnaire was ascertained using an evaluation form to help respondents assess each item in terms of ease of understanding the items by the target population, clarity, and essentiality of the items. For this process, a different cohort of 20 practitioners with clinical dental practice (with or without specialization), experience of a minimum of one year, and who were willing to participate were randomly selected and were asked to respond to the constructs indicated above. It was ensured by the investigators that the 20 practitioners selected were entirely different from the experts who had previously administered the questionnaire and were not to be included in the sample that was recruited for pilot testing. The practitioners were asked to rate the items of the modified survey based on their readability and clarity on the following scale: 1—not at all, 2—a little, 3—moderately, 4—very much, and 5—extremely.

#### 2.3.3. Phase III

A web-based survey was conducted from 3 February 2021 to 20 February 2021 using the newly developed survey tool. The survey was administered using the Qualtrics platform and only one response per participant was allowed to prevent multiple submissions. 

#### 2.3.4. Phase IV

Construct validity or item purification by exploratory factor analysis (EFA): The objective of this phase was to perform dimension reduction and to assess the robustness of the intended items included in the questionnaire. Before EFA, assumptions such as the Kaiser–Meyer–Olkin (KMO) measure of sample adequacy and Bartlett’s test of sphericity were tested [35]. The EFA was achieved using a traditional unweighted least squares extraction method through a varimax rotation with Kaiser normalization [36]. This approach was selected given the small sample size used in this study [36]. The EFA was run on a 28-item questionnaire that measured the compliance of Indian dentists towards DCI guidelines to minimize COVID-19 transmission. The EFA helped us to identify the underlying relationship between the measured items that constitute the constructs. The inter-factor correlation was assessed to examine whether factors were strongly correlated with each other. In such instances, we reran the EFA with the correlated factors to see if the items were correlated with both factors. To ensure discriminant validity, we removed items related to both factors. The extraction of the factors was not only informed by the factor loadings, but also by the interpretability of the factors. Items with low factor loading <0.40 and cross loading with differences below 0.2 were removed at each EFA iteration [37]. The final factor solution was obtained based on the number of eigenvalues greater than one [38] and a visual inspection of the scree plot [39]. The reliability of the questionnaire was assessed using Cronbach’s alpha [40,41]. As cited in the previous literature, the value of Cronbach’s alpha was deemed acceptable if it ranged from 0.70 to 0.90 [42].

### 2.4. Data Analysis

Given the pilot nature of this study, we used the flat sample size rule of sample size recommendations provided by previous simulation and trial studies. After accounting for the appropriate adjustment factor of 0.5 involving a single group only, the recommended pilot sample size ranged from 30 to 70 subjects, which aligned well with the sample used in this study [43,44,45,46]. However, this sample might not have been adequate for the factor analysis, due to which we used an unweighted least squares method suggested by Jung in 2012. Categorical variables were represented as frequencies and proportions, whereas continuous variables were represented by means and standard deviations. The normal approximation of the binomial distribution method was used to calculate 95% confidence intervals of proportions. All analyses were conducted using SPSS version 27 (Armonk, NY, USA: IBM Corp.).

## 3. Results

### 3.1. Evaluation by Expert Review

Content Validity: Based on the values obtained for I-CVI from round one of the content validity assessment, 27 items were retained. Three items (“How often do you defer patients for a cosmetic or a non-emergency dental treatments?”, “When a patient with COVID-19 symptoms is identified, how often do you refer the patient to a physician and recall the patient after clearance from the physician?”, and “How often do you take a shower/bath on reaching home after the dental clinic?”) that reported I-CVI scores of 0.57 were removed. Two items (“How often do you make cashless transactions in your clinic?” and “What is the frequency of paperless form of practice in the day-to-day work of your clinic?”) with I-CVI values between 0.71 and 0.85 were modified according to the recommendations given by the experts regarding “How often do you encourage payment of dental services in a cashless mode/electronic transfer?” and “How often do you encourage paperless form of practice in the day-to-day work of your clinic?”. All of the remaining items were found to have an acceptable score of I-CVI (0.83—1.00) [38,46,47,48]. On the basis of suggestions received from the experts, two items in reference to waste disposal, as per the guidelines, were added. 

The second round of the content validity assessment reported appropriate I-CVI values (0.83–1.00) for all items except one (“How often do you schedule the appointments of the patients exclusively telephonically?”), which showed a score of 0.6 and thus was excluded. In total, 86% of the items reported a ratio of between 0.8 and 1.00 with an average CVR of 0.77. The S-CVI values were reported as 0.91. The inter-rater reliability was calculated as 0.58 (sig.0.00). Thus, the results of the content validity assessment resulted in a pool of 28 items.

Face Validity: Using a five-point Likert scale, responses that rated items with four or five points were considered acceptable [49]. The majority (96%) of the practitioners indicated that the questions were clearly understood and were easy to answer and 100% indicated that they were satisfied with the overall layout and style of the questionnaire.

### 3.2. Construct Validity (Exploratory Factor Analysis)

**Figure 1:**
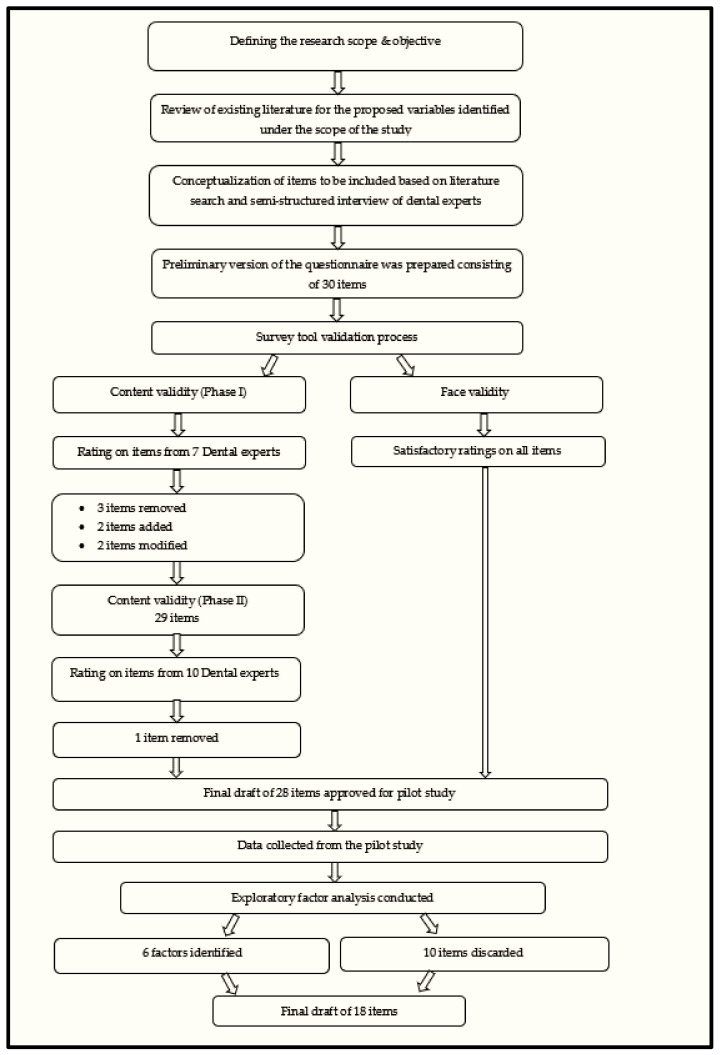
Flow chart depicting the design, validation, and pilot testing of the questionnaire.

Inspection of the correlation matrix showed that all variables (except one) had at least one correlation coefficient greater than 0.3. The overall Kaiser–Meyer–Olkin (KMO) measure was 0.63, which can be classified as “Mediocre” according to Kaiser [50]. Bartlett’s test of sphericity was statistically significant (*p* < 0.001), indicating that the data were likely factorizable. The EFA revealed six factors that had eigenvalues greater than one and which explained 30.2%, 10.5%, 9.8%, 8.3%, and 6.4% of the total variance, respectively. A six-factor solution was chosen as the final structure based on factor loadings, a visual inspection of the scree plot [51], and conceptual knowledge. The six-factor solution explained 72.9% of the total variance. A Varimax orthogonal rotation was employed to aid interpretability [52]. The rotated factor matrix is shown in Table 1. The sequential development of the final 18-item questionnaire from a 30-item questionnaire pool is described in Figure 1.

The resulting domains were interpreted by assigning them a name based on the original variables included in each domain. This confirmed that the six constructs underlying the 18-item instrument were screening (items 1 and 2), regular infection prevention measures (items 3–5), infection control inside the dental operatory (items 6–9), disinfection of the dental unit (items 10–13), disposal (items 14 and 15), and other COVID-19-specific preventive measures (items 16–18). 

### 3.3. Reliability Diagnostics

The global Cronbach’s alpha for the resulting questionnaire was 0.86. The Cronbach’s alpha values were 0.80 for screening, 0.84 for regular infection prevention measures, 0.71 for infection control inside the dental operatory, 0.88 for disinfection of the dental unit, 0.68 for disposal, and 0.67 for other COVID-19-specific preventive measures.

### 3.4. Demographic Characteristics of the Survey Respondents

Of the 64 participants, after excluding incomplete responses (*n* = 11) and those who did not provide their consent (*n* = 8), a total of 45 valid responses were analyzed. Over ¾ of the sample was aged between 25 and 39 years. With regard to the highest qualification, the sample was nearly equally split between those who possessed a Bachelor of Dental Surgery (BDS) or Master’s in Dental Surgery (MDS) degree. Over 50% of respondents rated DCI guidelines as good and 60% of the respondents self-rated themselves good at implementing the DCI guidelines at their workplace (dental operatory). The additional characteristics of the respondents can be seen in Table 2. 

As indicated in Table 3, the item-wise analysis revealed highest mean scores in the domains of compliance with regard to providing alcohol-based hand scrubs, providing protective gear to attendees, collecting travel/medical history, and screening patients for COVID-19 symptoms. In contrast, less compliance was observed regarding the use of paperless forms of practice and rubber dams in the operatory.

## 4. Discussion

The current study demonstrated the process of developing a new instrument to evaluate the adherence of dental practitioners to the guidelines provided by the DCI. Previous studies analyzed the knowledge, preparedness, and attitude of dental clinicians towards the COVID-19 pandemic; however, to the best of the authors’ knowledge, a survey tool to measure the uptake of the recommended guidelines (instituted during the COVID-19 pandemic) by dental clinicians is currently lacking [53,54,55,56]. 

Although the newly formed questionnaire was essentially developed to evaluate adherence to guidelines issued regarding COVID-19-related dental clinical protocols, the majority of the items are related to infection control measures, which might also be adapted to limit other nosocomial infections in the future. This questionnaire involved a rigorous methodology for the validation and will serve as a useful tool to measure the effectiveness of regulatory guidelines at individual and institutional levels. Health organizations, institutions, and practitioners will be able to effectively measure adherence to the issued guidelines by the DCI in the context of the COVID-19 pandemic or any diseases with similar routes of transmission. 

As the pandemic progressed in its course and the research landscape evolved, aiding researchers in gaining a better understanding of the epidemiology of the virus, restrictive measures across the globe were relaxed and modified. We then entered into a phase of the “New Normal,” which changed the methods of dental practice and strived to achieve a balance between safety and continuity of business operations. To ensure the safety of patients and dental practitioners, the governing dental body in India, the DCI, released its first advisories on COVID-19 guidelines for dental colleges and dental professionals on 16 April 2020, followed by the provision of a dental clinic protocol on 7 May 2020 [57]. Later, the Ministry of Health and family welfare released the National Guidelines for Safe Dental Practice during COVID-19 pandemic on 29 September 2021 [58]. The effectiveness of these guidelines and recommendations in curbing the spread of infection was largely dependent on their uptake. One study found that despite a large percentage of dentists (90%) being aware of changes made in the treatment protocols, the actual implementation of the amended treatment protocols in their clinical environment or operatories was only 61% [59]. 

With the help of the survey tool developed in the current study, adherence towards DCI guidelines can be measured and data can serve as a baseline to design targeted interventions. The results of the study showed that there were differences in various item scores, indicating that certain aspects of the guidelines were being followed better than others. It was found that there was a relatively greater uptake of guidelines related to screening for COVID-19 symptoms at the entrance of patients to the clinic, the collection of relevant travel and medical history, and the cleaning or removal of toys and reading materials. This finding was consistent with a previous study—where data were collected between 10 March and 17 March 2020—that involved dental surgeons from multiple countries, in which over 80% of dental surgeons preferred recording patients’ travel history [59]. This indicates our sample was knowledgeable about the risk factors and potential routes of transmission associated with COVID-19 infection [60]. Consistent with another study, the results of the current study revealed a high score regarding disposal of N95 masks and other disposable PPE in a red bin handed to an authorized biomedical disposal agency. A study by Raghuwanshi and others showed that a high percentage (76%) of the study respondents had knowledge of color-coded bins, and concluded that most of the dentists in North India had adequate knowledge regarding the Biomedical Waste (BMW) and related policies [61,62,63].

Items related to flushing the pipelines with a disinfectant, flushing dental unit waterlines with a disinfectant, and flushing the suction pumps with a disinfectant before and after a procedure obtained low mean scores. Similarly, items on the use of PPE (for non-aerosol procedures), the use of dedicated bins/trolleys or bags for transporting waste to a site, the use of fumigation, and the use of rubber dams for preventing cross-infections received lower mean scores. These findings were consistent with the results of a study by Shenoy and others, who found that a lower proportion (35.9%) of their participants did not use rubber dams [54]. The poor use of rubber dams by dentists in India has been reported previously, where the lack of training, time taken for placement, and added cost represented the reported barriers to their use [64]. Another study found that that 76.2% of dentists did not use a rubber dam in their routine clinical practice, while 40% of dentists found the use of rubber dams time consuming, 37% did not use them due to patient compliance, 14% responded that they are expensive, and 9% were unsure of the technique [65]. Providing dental clinicians with training focused on improving the required skills and attitudes towards the effective use of rubber dams has been suggested to improve their usage [66]. Regarding paperless forms of dental practice, they are not being practiced largely, especially in smaller dental clinics in India. The use of electronic patient records, tele dentistry, and cashless modes of payment largely compose paperless forms of practice [67]. Though the use of electronic health records and tele dentistry have largely increased in India, the utilization of these on a regular basis in smaller dental clinics may not be prevalent. Further research must be undertaken to understand the adoption of such practices as a response to the pandemic.

The results of the pilot study showed the highest mean scores for items related to providing alcohol-based hand scrubs in the clinic, followed by items on providing gloves/masks/a change of clothes to attendees. The importance of hand hygiene cannot be underestimated. In a recent publication, European oral surgery specialists provided their consensus with regard to the significance of hand hygiene to decrease the risk of COVID-19 transmission in dental settings [68]. The results of the study were consistent with a previous study, which indicated that 99.5% of dentists advocated hand washing and alcohol rubs in their clinical practice, but encouraging when compared to other studies, where it was found that compliance with hand hygiene was not very high, ranging from 35 to 56% [56,69,70,71].

The current study highlights the importance of continuing adherence to the guidelines as the COVID-19 pandemic continues and remains a public health threat, even in its third year of its existence. As of 8 June 2022, there have been 530,896,347 confirmed COVID-19 cases, including 6,301,020 deaths, as reported by the WHO [72]. Further, recently identified, highly transmissible, and evolving variants—such as B.1.1.7 (202012/01), B.1.351 (501Y.V2), and P.1.—continue to draw the attention of health authorities [73,74]. This rise in cases could be the fallout of the relaxation of public health measures, such as the reluctance to adhere to preventive social measures, including social distancing, avoiding crowds, wearing of masks, and hand hygiene [75,76]. Given the observed rise in the number of cases, it can be said that the pandemic is far from over and effective preventive measures must continue to be followed, especially in dental care settings. 

Various reasons can be attributed to the results obtained in the current study. Firstly, the obtained results reflect the knowledge and training in infection control practices, as the adherence to guidelines is said to be influenced by knowledge and attitudes towards COVID-19. Further, dental practitioners’ knowledge and attitudes provide insights into the perceptions and practices adopted as responsive behaviors to the pandemic [77]. The fact that more items received a higher score is indicative of the knowledge and attitude of dental practitioners in India [78,79]. The conduction of regular training programs and reinforcement of the guidelines by healthcare authorities are some of the known methods of enhancing the knowledge and awareness among healthcare professionals. During the pandemic, effective measures were taken by the health authorities in India to ensure the spread of COVID-19-related knowledge among practitioners. Online webinars on COVID-19 disease and infection control protocols were conducted by the DCI and were attended by more than 10,000 dentists from across the country [80]. 

While certain guidelines received a good response, a few others were not being so well adhered to. This could be related to the cost incurred, directly or indirectly, when following the imposed guidelines, as dentists may have found their implementation economically less viable [81]. One of the known consequences of COVID-19 is the change in the manner of dental practice, and some changes are said to be costlier, more cumbersome, and time consuming [82]. A study conducted on 500 Indian dentists revealed that 92.3% of dentists perceived monetary investments to be a major barrier to safe dental practice [82]. This could be a possible reason for the poor response obtained for some of the items, in which additional expenditure was likely to be required for implementation of the guidelines. 

Another reason for the poor response in certain items observed could be related to the source and flow of misinformation related to the guidelines. During the pandemic, various countries published different guidelines, some being contradictory, making information clarity challenging for dentists. A recent review found that recommended guidelines had not been successfully implemented in spite of an awareness of the risks involved [83]. Further, not all practicing dentists may have received the information from a reliable information source. Though sources such as social media and the internet provide information that is accessible at all times, the information provided by these sources may not always be accurate [55]. Lastly, different states or districts across the country peaked at different times, which may have affected both the preventive measures implemented by a particular region in a given timeframe and the results obtained [82,83].

In the current study, a conceptual framework and subsequent items were developed based on a thorough literature search and interviews, followed by a robust validation process and pilot testing. This finally resulted in the development of a tool with relevant items and domains essential for preventing the spread of COVID-19 infection during dental practice in India. The applications of this newly developed tool are manifold. First, it can be used to assess the adherence of dental clinicians to certain guidelines. Second, the tool can further be used to monitor the impact of awareness programs by the DCI for infection control during the pandemic. Finally, it may be useful for policymakers in India to help identify barriers and develop strategies to improve adherence to the guidelines. With regard to the future applicability of the existing tool, COVID-19 items may be of less interest in the post-pandemic period. However, the tool will still offer a wide range of adaptability to other diseases with a similar epidemiology. Nevertheless, this study is not without limitations. First, due to the small sample size, we could not investigate subgroup differences by demographic variables. Moreover, its generalizability would be questionable given the small sample size. Future studies should be planned with a larger sample size, which will also allow for confirmatory factor analysis to validate the factor solution obtained through the exploratory factor analysis used in the current study. Second, there were some variables that were left unmeasured, for instance, the type of dental practice setting (private/semi-private/government), workplace (hospital vs. clinic), years of experience, daily workload, etc. Future studies should be planned to measure differences in compliance according to such variables in order to understand the entire breadth of the problem. Finally, the results of the study might have been impacted by social desirability bias and non-response bias. 

## 5. Conclusions

The assessment of adherence to dental practice guidelines can help clinicians and oral health authorities take effective measures to improve efforts towards the prevention of the spread of infection while continuing to maintain dental practice. The results obtained from using this tool can be used by health policymakers to identify specific barriers and develop strategies to improve adherence to dental practice guidelines. The use of a validated survey tool ensures the collection of reliable and valid data, which can serve as baseline data to measure the uptake and effectiveness of dental practice regulatory guidelines in clinical settings and community dental health clinics.

## Figures and Tables

**Table 1:** Factor rotation matrix revealed by exploratory factor analysis.

Factor Rotation Matrix (a)	Factor 1	Factor 2	Factor 3	Factor 4	Factor 5	Factor 6
Item 1				0.650		
Item 2				0.755		
Item 3		0.898				
Item 4		0.644				
Item 5		0.871				
Item 6			0.759			
Item 7			0.578			
Item 8			0.602			
Item 9			0.408			
Item 10	0.753					
Item 11	0.821					
Item 12	0.521					
Item 13	0.876					
Item 14					0.640	
Item 15					0.773	
Item 16						0.667
Item 17						0.603
Item 18						0.474

Extraction method: Unweighted least squares. Rotation method: Varimax rotation with Kaiser standardization; (a) six-factor solution.

**Table 2:** Summary statistics of the sample (*n* = 45).

Variable	Groups	*n* (%)	95% CI of Proportion
Gender	Male	29 (64.5)	48.7, 78.1
	Female	16 (35.5)	21.8, 51.2
Age range	25–39 years	35 (77.8)	62.9, 88.8
	40–49 years	9 (20.0)	9.6, 34.6
	50–59 years	1 (2.2)	0.6, 11.7
Highest qualification	BDS	21 (46.7)	31.6, 62.1
	MDS	20 (44.4)	29.6, 60.0
	P.G. Diploma	4 (8.9)	2.5, 21.2
Rating of DCI guidelines reported by the respondents	Excellent	11 (24.4)	12.8, 39.5
	Good	23 (51.1)	35.7, 66.3
	Average	7 (15.6)	6.5, 29.5
	Fair	3 (6.7)	1.4, 18.3
	Poor	1 (2.2)	0.6, 11.7
Self-rating in implementing the guidelines by the dentists	Excellent	4 (8.9)	2.5, 21.2
	Good	27 (60.0)	44.3, 74.3
	Average	9 (20.0)	9.6, 34.6
	Fair	4 (8.9)	2.5, 21.2
	Poor	1 (2.2)	0.6, 11.7

CI: Confidence interval.

**Table 3:** Assessing adherence towards DCI guidelines based on the final 18-item questionnaire (*n* = 45).

Domain or Construct	Items	Mean ± SD
Screening	In your clinic how often are all patients screened for COVID-19 symptoms at the entry of the clinic?	4.46 ± 0.92
	How often do you take relevant travel and medical history for each patient in your clinic?	4.46 ± 0.94
Regular infection prevention measures	How often do you provide alcohol-based hand rubs for the patients in your clinic?	4.77 ± 0.71
	How often are the toys, reading materials, remote controls or other communal objects removed or cleaned regularly in your clinic?	4.26 ± 0.98
	How often do you provide gloves/mask/change of clothes to your attenders in the clinic?	4.73 ± 0.75
Infection control inside the dental operatory	How often do you use rubber dams (where ever indicated) in your dental practice?	2.44 ± 1.34
	How often do you use high volume saliva ejectors in your practice?	3.28 ± 1.55
	How often do you provide shoe covers to your patients?	3.51 ± 1.51
	After completion of the procedure, how often do you dispose of the N 95 mask and another disposable PPE in a red bin to be handed to an authorized biomedical disposal agency?	4.06 ± 1.16
Disinfection of dental unit	After a dental procedure, how often do you ensure that the dental unit waterlines (DUWL) are flushed, disinfected using appropriate organic disinfectant and are drained? (Applicable to units which do not have non-retraction valves).	3.68 ± 1.14
	After a dental procedure, how often do you ensure suction pumps are flushed with chemical cleaning solution as per manufacturer’s instructions?	3.86 ± 1.05
	Before each procedure, how often do you ensure that the suction pumps are flushed with a chemical cleaning solution?	3.77 ± 1.20
	How often do you flush the pipelines with an appropriate disinfectant?	4.00 ± 1.01
Disposal	How often do you use disposable gowns for non-aerosol producing dental procedures?	3.55 ± 1.42
	How often do you use dedicated collection bins/trolleys/bags labeled as COVID-19 for transporting waste from the clinical area to the disposal site?	3.56 ± 1.47
Other COVID-19-specific preventive measures	After a dental procedure, how often do you fumigate the operatory and leave it unused for 30 min?	3.66 ± 1.06
	How often do you measure the body temperature and use a pulse oximeter to screen patients for COVID-19 symptoms?	4.44 ± 0.91
	How often do you encourage paperless forms of practice in the day-to-day work of your clinic?	2.95 ± 1.30

## Data Availability

The data presented in this study are available on request from the corresponding author. The data are not publicly available due to ethical reasons.
